# Simultaneous detection of novel H7N9 and other influenza A viruses in poultry by multiplex real-time RT-PCR

**DOI:** 10.1186/s12985-015-0300-x

**Published:** 2015-04-30

**Authors:** Xiaolong Xu, Hongmei Bao, Yong Ma, Jiashan Sun, Yuhui Zhao, Yunhe Wang, Jianzhong Shi, Xianying Zeng, Yanbing Li, Xiurong Wang, Hualan Chen

**Affiliations:** State Key Laboratory of Veterinary Biotechnology, Harbin Veterinary Research Institute, Chinese Academy of Agricultural Sciences, Harbin, 150001 People’s Republic of China

**Keywords:** Multiplex rRT-PCR, H7N9 virus, Influenza A virus

## Abstract

**Background:**

A novel reassortant H7N9 influenza A virus has crossed the species barrier from poultry to cause human infections in China in 2013 and 2014. Rapid detection of the novel H7N9 virus is important to detect this virus in poultry and reduce the risk of an epidemic in birds or humans.

**Findings:**

In this study, a multiplex real-time RT-PCR (rRT-PCR) assay for rapid detection of H7N9 and other influenza A viruses was developed. To evaluate the assay, various influenza A viruses, other avian respiratory viruses, and 1,070 samples from poultry were tested. Fluorescence signals corresponding to H7 and N9 subtypes were detected only when H7 and N9 subtypes were present, while the fluorescence signal for the influenza A M gene was detected in all specimens with influenza A strains. The fluorescent signal can be detected in dilutions as low as 56 copies per reaction for the H7, N9 and M genes. Intra- and inter-assay variability tests showed a reliable assay. In poultry samples, a comparison of rRT-PCR with virus isolation showed a high level of agreement.

**Conclusions:**

The multiplex rRT-PCR assay in this study has good specificity, sensitivity and reproducibility, and will be useful for laboratory surveillance and rapid diagnosis of H7N9 and other influenza A viruses.

## Findings

From 1996 to 2014, human infections with H7 subtypes (H7N2, H7N3, and H7N7) have been reported in many regions of the world. Most of these infections occurred in association with poultry outbreaks, and the infections mainly resulted in conjunctivitis and mild upper respiratory symptoms, with the exception of one death, which occurred in a veterinarian in the Netherlands [1-4]. Until March 2013, no human infections with H7N9 virus had been reported.

On 31 March 2013, the National Health and Family Planning Commission of China announced that human infections with H7N9 virus had occurred in Shanghai and Anhui Province, China. Because this novel reassortant H7N9 virus had not previously been detected in animals or humans, the situation has raised urgent questions and global public health concerns [5]. As of 14 July 2014, 451 human infections have been identified [6]. Some of the cases of human infections with this H7N9 virus have reported exposure to live poultry or to potentially contaminated environments, particularly markets where live birds have been sold [7].

H7N9 influenza does not kill poultry, which makes surveillance in poultry much more difficult. Therefore, it is critical to identify the H7N9 virus rapidly in different types of poultry samples to control potential transmission to humans. Conventional laboratory methods for H7N9 virus detection currently include virus isolation in embryonated eggs, followed by HA and NA subtype identification by serological methods such as hemagglutination and hemagglutination inhibition [8-10]. However, the emergence of this new H7N9 virus as a cause of severe illness and death in people requires testing in laboratories with higher biosafety levels than typical influenza viruses, and virus isolation of the H7N9 virus in embryonated eggs may increase the risk of infections in laboratory workers [10,11]. Hence, a rapid and sensitive molecular method for the identification of H7N9 virus is needed to facilitate clinical care and infection control.

Molecular detection methods such as rRT-PCR assays have been widely applied for the laboratory diagnosis of virus infections, as a result of their high sensitivity and rapid turnaround times [12,13]. After the emergence of the H7N9 virus, rRT-PCR methods for the detection of H7N9 virus have been developed [14-16]. In addition, the World Health Organization (WHO) Collaborating Center for Reference and Research on Influenza at the Chinese National Influenza Center, Beijing, China, has made an rRT-PCR protocol for the detection of H7N9 virus available [17]. However, these methods do not allow the simultaneous detection of both the HA and NA genes of the virus.

Here, we describe a multiplex rRT-PCR assay for simultaneously detecting H7N9 and other influenza A viruses with high specificity and sensitivity. Including the time required for RNA extraction, results can be obtained in 2.5 h with this assay, which will facilitate laboratory surveillance and rapid diagnosis of H7N9 and other influenza A viruses.

The nucleotide sequences of H7N9 viruses were obtained from sequences of viruses in our laboratory, and the H7 and N9 primers and probes were chosen based on the conserved regions of 52 sequences. Primers and probes were designed and analyzed using Lasergene (DNASTAR Inc., Madison, WI, USA) and Primer Express 3.0 (Applied Biosystems, Foster City, CA, USA). The M gene was selected for detection of influenza A virus, and the primer and probe set for the M gene was taken from WHO recommendations. The primers and probes used in this study are listed in Table 1. Two of the probes were labeled with BHQ1 at the 3′ end, another probe was labeled with BHQ3, and three different fluorescent reporter dyes (FAM, JOE, and Cy5) were at the 5′ ends to allow simultaneous detection of the three genes in a single reaction.

**Table 1:** **Primers and probes for the detection of H7N9 and other influenza A viruses**

**Name**	**Sequences (5′-3′)**
H7-Forward	CTAATTGATGGTTGGTATGGTTTC
H7-Reverse	AATTGCCGATTGAGTGCTTTT
H7-Probe	FAM-CAGAATGCACAGGGAGAGGGAACTGCT-BHQ1
N9-Forward	CAGAAGGCCTGTTGCAGAAATT
N9-Reverse	CCGTTGTGGCATACACATTCAG
N9-probe	JOE-CACATGGGCCCGAAACATACTAAGAACACA-BHQ1
M-Forward	GAC CRA TCC TGT CAC CTC TGA C
M-Reverse	AGG GCA TTY TGG ACA AAK CGT CTA
M-Probe	Cy5-TGC AGT CCT CGC TCA CTG GGC ACG-BHQ3

Viral RNA was extracted from samples using the QIAmp viral RNA mini kit (Qiagen, Valencia, CA, USA) according to the manufacturer’s instructions. The multiplex rRT-PCR was performed using the AgPath-ID™ one-step RT-PCR Kit (Ambion Inc., Austin, TX, USA) in a 20-μL reaction mixture containing 10 μL of 2× RT-PCR Buffer, 0.8 μL of 25× RT-PCR Enzyme Mix, 4 μL of RNA sample RNA, and three sets of primers and probes. The primers for H7, N9, and M genes were used at optimized concentrations of 0.6 μM, 0.6 μM, 0.7 μM, while the probes for H7, N9, and M genes were used at final concentrations of 0.4 μM, 0.4 μM, and 0.6 μM, respectively. The reaction was conducted with a LightCycler 480 Real-Time PCR System (Roche Inc., Basel, Switzerland). The rRT-PCR program included a reverse transcription step at 45°C for 30 min, a hold at 95°C for 10 min, and 40 amplification cycles of 95°C for 5 s and 60°C for 1 min. Fluorescent signals were evaluated during the elongation step.

**Figure 1:**
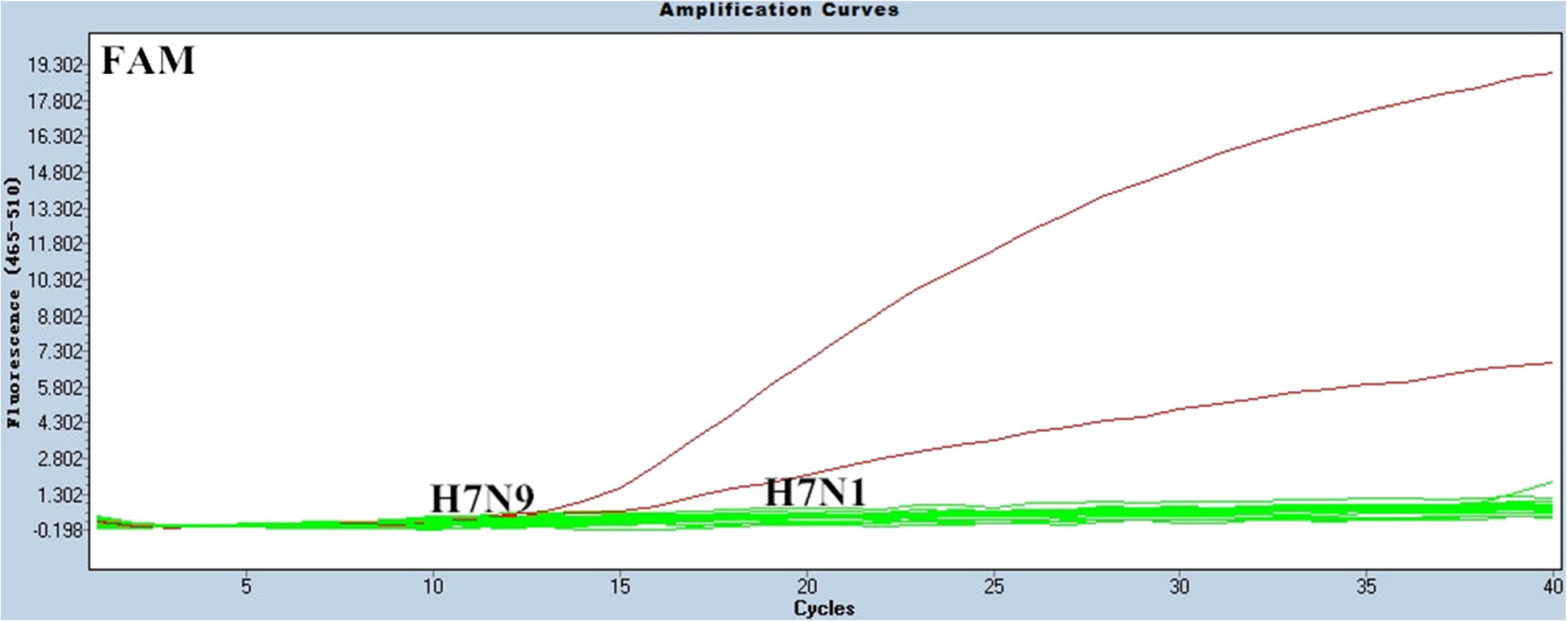
Specificity results of the rRT-PCR assay for the detection of the H7 gene. In the FAM channel, only the two H7 subtype viruses yielded positive curves.

**Figure 2:**
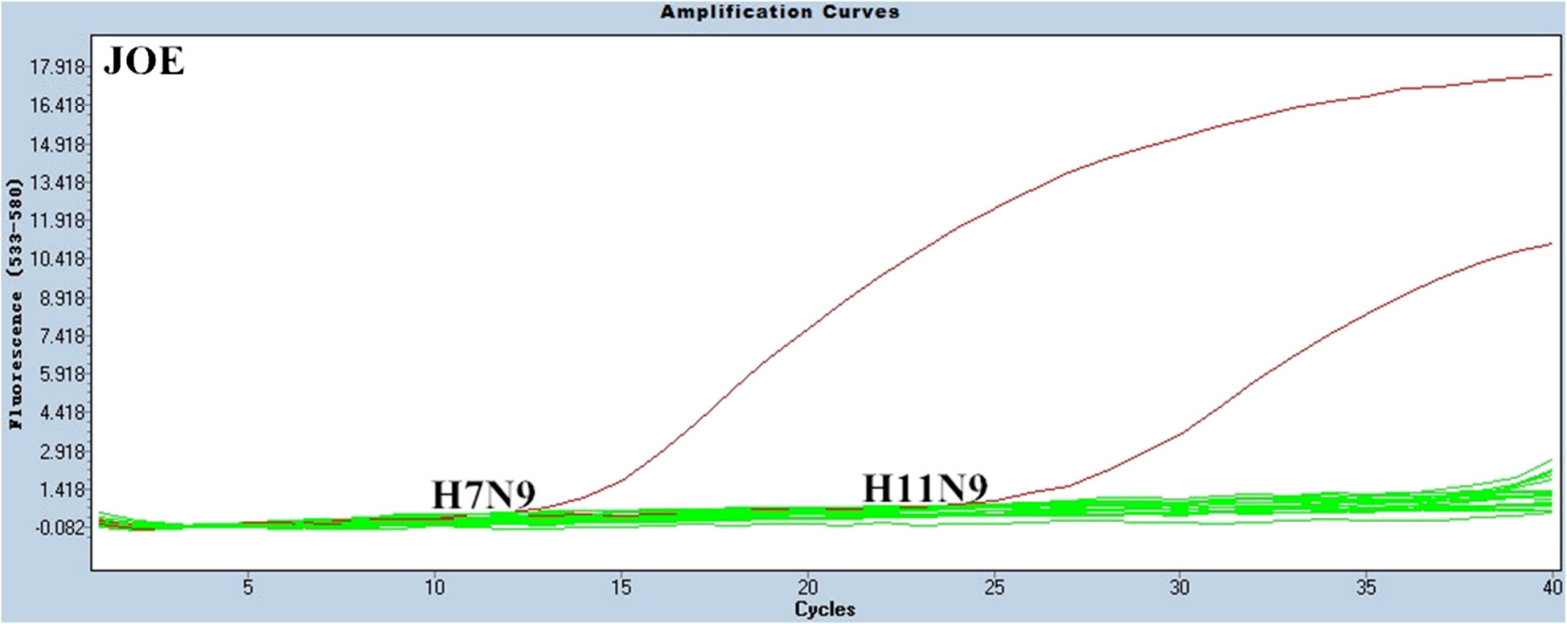
Specificity results of the rRT-PCR assay for the detection of the N9 gene. In the JOE channel, only the two N9 subtype viruses yielded positive curves.

**Figure 3:**
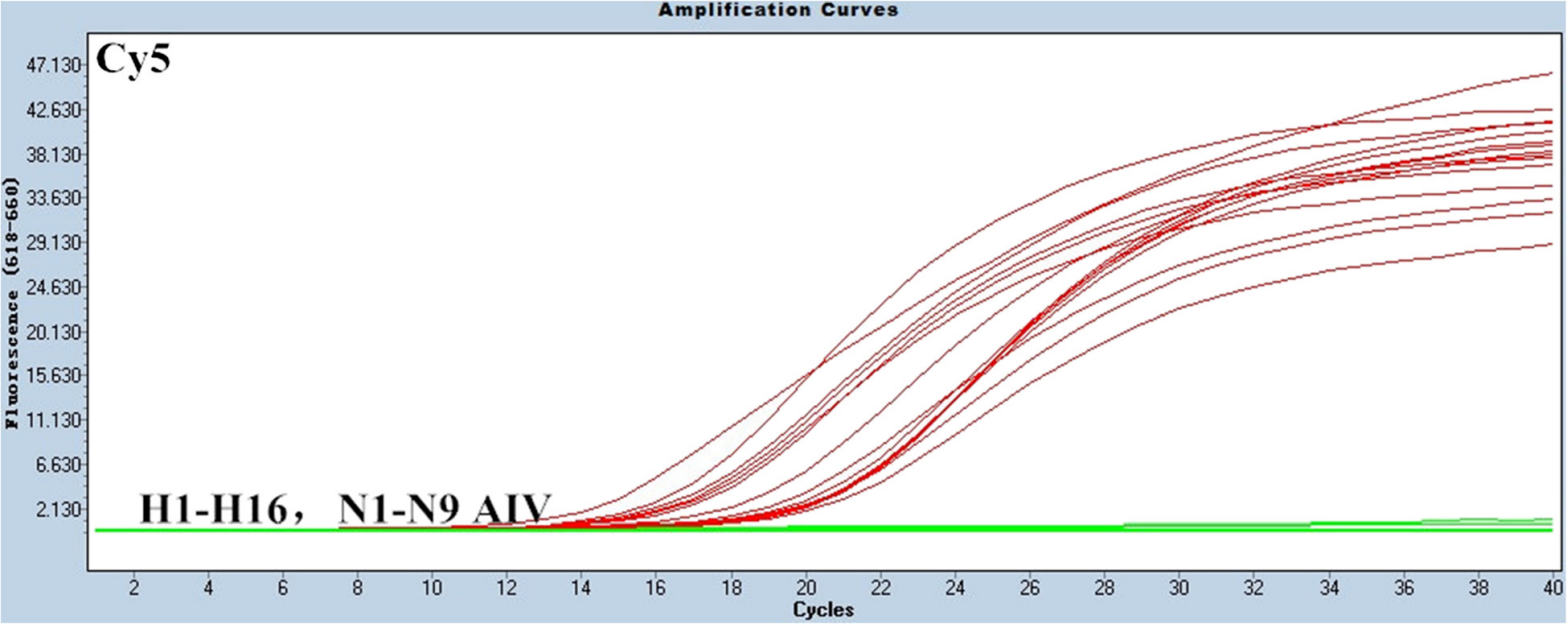
Specificity results of the rRT-PCR assay for the detection of influenza A. In the Cy5 channel, only the influenza A reference strains yielded positive curves.

The specificity of the multiplex rRT-PCR assay was evaluated using RNA samples that were extracted from H1-H16 and N1-N9 influenza strains and other avian respiratory viral pathogens, including Newcastle disease virus (NDV), infectious bronchitis virus (IBV), infectious bursal disease virus (IBDV) (Table 2), and non-inoculated allantoic fluid. All samples with H7 subtype viruses gave positive FAM fluorescence signals, as expected (Figure 1), and samples with N9 subtypes were positive for JOE fluorescence signals, also as expected (Figure 2). Moreover, all influenza A reference strains yielded the standard fluorescence signal in the Cy5 channel (Figure 3).

**Table 2:** **Specificity results of the multiplex rRT-PCR**

**Name of isolate**	**Virus**	**H7-FAM**	**N9-JOE**	**Flu A-Cy5**
A/mallard/Sanjiang/390/2007	H1N1	-	-	+
A/mallard/Heilongjiang/135/2006	H2N2	-	-	+
A/mallard/Heilongjiang/90/2006	H3N2	-	-	+
A/duck/Guangxi/S-2-248/2009	H4N6	-	-	+
A/duck/Guangdong/S1322/2010	H5N1	-	-	+
A/mallard/Heilongjiang/81/2006	H6N2	-	-	+
A/pigeon/Shanghai/S1069/2013	H7N9	+	+	+
A/AfricanStarling/England/983/1979	H7N1	+	-	+
A/turkey/Ontario/6118/1968	H8N4	-	-	+
A/turkey/Scotland/1970	H9N7	-	-	+
A/Turkey/England/384/1979	H10N4	-	-	+
A/Duck/Memphis/546/1976	H11N9	-	+	+
A/duck/Alberta/60/1976	H12N5	-	-	+
A/gull/Maryland/704/1977	H13N6	-	-	+
A/mallard/Gurjer/263/1982	H14N5	-	-	+
A/mallard/Gurjer/263/1982	H15N8	-	-	+
A/Cormorant/Denmark/74-68899-G2/2002	H16N3	-	-	+
NDV-1	LaSota	-	-	-
NDV-2	F48E9	-	-	-
IBV	Lx4	-	-	-
IBDV	Gt	-	-	-
Non-inoculated allantoic fluid		-	-	-

The detection limits of the assay were evaluated with serial 10-fold dilutions of RNA from A/pigeon/Shanghai/S1069/2013 (1.4 × 10^6^ viral genome copies/μL) ranging from 10° to 10^−8^. The fluorescent signal could be detected from the H7, N9, and M genes as low as 56 copies per reaction (10^−5^ dilution).

To evaluate the reproducibility of the assay, six replicates at the 10°, 10^−2^, and 10^−4^ dilutions were tested. The coefficient of variation was calculated to measure the inter- and intra-reproducibility of the assay. The coefficients of variation within a single run (intra-assay variability) ranged from 0.11% to 1.22%, whereas the coefficients of variation in different runs (inter-assay variability) ranged from 0.78% to 1.48%. The results showed good reproducibility (Table 3).

**Table 3:** **Intra- and inter-assay coefficients of variation for multiplex rRT-PCR**

**Dilution of RNA**	**n**	**Intra-assay (%)**	**Inter-assay (%)**				
		**H7**	**N9**	**M**	**H7**	**N9**	**M**
10°	6	0.97	0.96	0.91	1.48	1.38	0.14
10^−2^	6	0.49	1.22	0.58	0.80	0.79	0.78
10^−4^	6	0.38	0.32	0.11	1.01	1.34	0.98

To further evaluate the assay, 1,070 tracheal and cloacal swabs were tested. Among these samples, virus isolation identified 24 influenza A virus positive samples, seven of which were H7N9 viruses. Multiplex rRT-PCR identified influenza A virus in 23 samples, and seven of these were H7N9 viruses. A sequence of the sample which was culture positive but negative by rRT-PCR matched influenza A virus. RT-PCR identified 19 samples with influenza A, of which four were H7N9 (Table 4). These results show a high level of agreement between multiplex rRT-PCR and virus isolation, and indicate that the multiplex rRT-PCR assay was more sensitive and accurate than the RT-PCR method.

**Table 4:** **Detection of clinical samples by virus isolation, rRT-PCR and RT-PCR**

	**Virus isolation**	**rRT-PCR**	**RT-PCR**
Positive influenza A virus	24/1,070	23/1,070	19/1,070
Positive H7N9	7/1,070	7/1,070	4/1,070

The novel H7N9 virus that causes severe human respiratory infections was first identified in China [18]. Although the transmission of H7 subtype viruses to humans has been previously reported [19], this was the first time that an N9 subtype virus infected humans. Preliminary genetic analysis has shown that all genes of the novel H7N9 virus are of avian origin, and are recombined from H7, N9 and H9N2 influenza A viruses [20]. Currently, person-to-person transmission of the virus has not been reported, but there is a potential risk that mutations in the virus could facilitate this transmission [21].

Because the novel H7N9 virus can circulate in poultry without symptoms, much attention has focused on the monitoring and detection of this virus in poultry. In this study, a one-step multiplex rRT-PCR assay was developed to simultaneously identify H7N9 and other influenza A viruses. The one-step nature of the assay reduces the risk of carryover contamination. After repeated experiments, the results suggested that compared with single-probe rRT-PCR for each of the gene targets, multiplexing did not change the sensitivity of detection limits (data not shown). Specific probes labeled with different fluorescent dyes were used to distinguish H7N9 from other influenza A viruses. Only H7N9 viruses have triple fluorescent signals in this assay. In addition, H7 or N9 subtypes from other lineages can also be detected by this assay: if only H7 is detected while N9 is not, it indicates that an H7 virus with another NA subtype is in the sample. Likewise, if only N9 is detected while H7 is not, it means that an N9 virus with another HA subtype is in the sample. The limit of the detection of the assay is 56 copies per reaction for all three targeted genes.

After testing 1,070 avian samples, 23 were positive by rRT-PCR. The Ct values of these positive samples ranged from 25 to 28, and most of the Ct values of negative samples ranged >30. The rRT-PCR assay had >95% accuracy (Table 4). Based on the Ct values of the avian samples, and because the rRT-PCR assay was developed to detect clinical samples in poultry, we recommend that a Ct ≤30 be regarded as positive. If the Ct lies between 30 and 32 and an obvious amplification curve is suggested, the test should be repeated, and the sample is considered positive if the repeated result is the same as before. If the initial Ct is between 30 and 32 and the repeat test is above 32, the sample should be tested with another method such as sequencing.

In conclusion, a sensitive and specific multiplex rRT-PCR assay for the detection of H7N9 and other influenza A viruses was developed and evaluated in this study. Compared with RT-PCR and virus isolation, the rRT-PCR assay was more rapid. Obtaining results within 2.5 h is important for the rapid detection of H7N9 viruses. We hope that this accurate and rapid diagnostic method will contribute to the prevention and detection of H7N9 and other influenza A viruses.
